# Recent Developments in Computed Tomography for Urolithiasis: Diagnosis and Characterization

**DOI:** 10.1155/2012/606754

**Published:** 2012-08-16

**Authors:** P. D. Mc Laughlin, L. Crush, M. M. Maher, O. J. O'Connor

**Affiliations:** Department of Radiology, Cork University Hospital and University College Cork, Cork, Ireland

## Abstract

*Objective. *To critically evaluate the current literature in an effort to establish the current role of radiologic imaging, advances in computed tomography (CT) and standard film radiography in the diagnosis, and characterization of urinary tract calculi. *Conclusion*. CT has a valuable role when utilized prudently during surveillance of patients following endourological therapy. In this paper, we outline the basic principles relating to the effects of exposure to ionizing radiation as a result of CT scanning. We discuss the current developments in low-dose CT technology, which have resulted in significant reductions in CT radiation doses (to approximately one-third of what they were a decade ago) while preserving image quality. Finally, we will discuss an important recent development now commercially available on the latest generation of CT scanners, namely, dual energy imaging, which is showing promise in urinary tract imaging as a means of characterizing the composition of urinary tract calculi.

## 1. Introduction

Since its first description by Smith et al. in 1995, noncontrast computed tomography (CT) of the urinary tract has become the imaging investigation of choice in patients with acute renal colic [1–3] due to its significantly higher sensitivity and specificity for detection of urinary stones when compared with plain radiography and intravenous urography and also due to superior capability for accurate characterization of the size and location of obstructing urinary calculi, thus allowing clinicians to predict the likelihood of spontaneous passage [4, 5].

Although CT is extremely valuable in imaging residual calculi and small fragments following endourological treatment, there are accepted limitations inherent to the technique, which are important in this setting.

1. Limited spatial resolution: CT has limited spatial resolution; therefore, its negative predictive value in fully excluding submillimeter calculi and small stone fragments is significantly less than its negative predictive value in excluding larger calculi (>4 mm).
2. Use of ionizing radiation: patients may present with their first episode of renal colic at a young age, and recurrence in subsequent years is reported in 25–50% of cases [6–8]. Repeated use of CT in these patients can result in a substantial cumulative dose; Ferrandino et al. reported a large cumulative effective radiation dose attributable to CT scanning during short-term followup of patients with urinary calculi [9]. Therefore, judicious and informed use of this modality is mandatory when imaging residual stones following endourological therapy in an effort to minimize the potential for significant exposures to ionizing radiation.

Despite these challenges, CT has a valuable role when utilized prudently during surveillance of patients following endourological therapy. In this paper, we outline the basic principles relating to the effects of exposure to ionizing radiation as a result of CT scanning. We discuss the current developments in low-dose CT technology, which have resulted in significant reductions in CT radiation doses (to approximately one-third of what they were a decade ago) while preserving image quality. Finally, we will discuss an important recent development now commercially available on the latest generation of CT scanners, namely dual energy imaging, which is showing promise in urinary tract imaging as a means of characterizing the composition of urinary tract calculi.

## 2. Ionizing Radiation

One of the most feared adverse events associated with exposure to ionizing radiation is carcinogenesis, which is a stochastic effect, that is to say, it is random. Cancer induction does not exhibit an upper or lower threshold of occurrence, and the probability of cancer induction is variable [10]. The overall risk of cancer is currently believed to be small, but a cause for concern with regard to radiation exposures in the diagnostic imaging range is that there is no radiation dose below which cancer induction does not occur. In addition, it is important to highlight that carcinogenesis may transpire many years following exposure, and it is accepted that exposure to ionizing radiation in early life magnifies the risk of tumor induction [11].

The general population is becoming increasingly aware and concerned about the potential health risks associated with ionizing radiation [12]. This is due to recent widespread press and media coverage highlighting the potential increase in cancer risk from exposure to ionizing radiation related to the increasing use of CT [13]. Today's young and educated patients with renal colic may in particular be conscious of exposure to ionizing radiation as a result of diagnostic imaging, and cancer induction is their primary concern. Their apprehension is not unfounded as large cumulative effective doses of >50 mSv during short-term followup (<1 year) were reported by Ferrandino et al. in 20% of patients with renal colic [9], and previous studies have suggested that cumulative exposure to similar levels of ionizing radiation (75 mSv) from sources other than CT may increase mortality due to cancer by 7.3% [11].

## 3. Low-Dose CT: An Update

Many studies have examined the diagnostic efficacy of low-dose CT in the setting of renal colic, and a variety of protocols have described that result in effective radiation dose reductions of up to 95% from greater than 10 mSv to as low as 0.5–3.5 mSv [14–24]. Low-dose CT is uniformly associated with an increase in image noise, but successes in dose reduction in the setting of renal colic have been aided by the inherent high contrast of renal calculi against the relatively low-density soft tissues surrounding the urinary tract.

**Figure 1:**
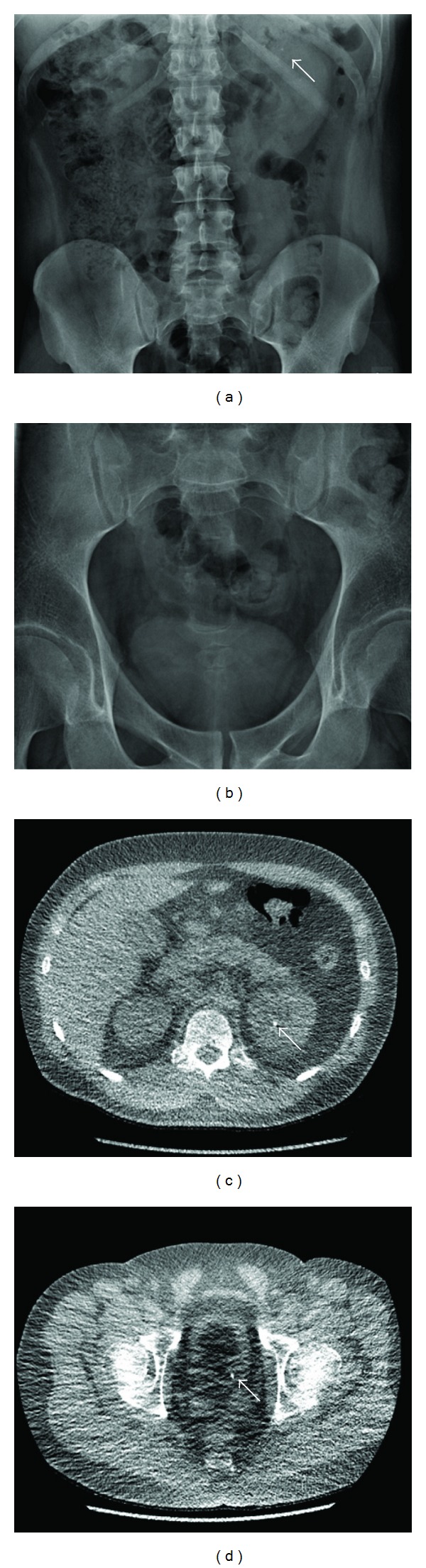
A 25-year-old male presenting with left flank pain. (a) Plain radiograph of the abdomen showing a possible renal calculus in the upper pole of the left kidney (estimated institutional dose ~0.7 mSv). The coned pelvic radiograph (b) does not demonstrate a calculus in the pelvis. Low-dose CT KUB (effective dose of 0.5 mSv) (c) and (d) clearly identifies a 5 mm calculus in the upper pole of the left kidney, as well as a 4 mm calculus, at the left ureterovesical junction. The latter was not seen on the plain radiograph.

In the past, fixed CT settings (e.g., kVp and mAs) resulted in lower attenuating areas such as the midabdomen receiving the same radiation exposure as higher attenuating regions such as the pelvis. This was an inefficient method of image acquisition and of imparting radiation exposure to patients as some regions were overirradiated, without any benefit in terms of image quality, while other regions were potentially underexposed, increasing image noise and reducing image quality. Automatic tube current modulation (ATCM) was a major development in CT technology in the last decade. One of the first major papers which evaluated ATCM as a means of optimizing radiation dose at CT reported dose reductions of 32% in 87% of CT examinations using ATCM [25]. Reductions in CT dose inherently create an increase in image noise, and the current focus of research and development in the area of CT radiation dose optimization is on the development of noise reduction algorithms to help preserve image quality in CT images acquired at a significantly reduced radiation dose; iterative reconstruction algorithms currently represent the most exciting dose optimizing developments in CT [26] (see Figure 1). Various modifications of iterative reconstruction are being developed and refined by different CT manufacturers including: adaptive statistical iterative reconstruction (ASIR) (General Electric Healthcare, Milwaukee, Wisconsin), sinogram affirmed iterative reconstruction (SAFIRE) (Siemens Healthcare, Erlangen, Germany), iterative reconstruction in image space (IRIS) (Siemens Healthcare, Erlangen, Germany), adaptive iterative dose reduction (AIDR) (Toshiba Medical Systems, Tustin, California), and iDose (Phillips Healthcare, Best, The Netherlands).

Emerging iterative reconstruction algorithms are typically noise efficient and computationally fast, and studies to date have mostly found images with good low-contrast detail, preserved image quality, and have facilitated dose reductions of between 20 and 60% in a variety of phantom [27–31] and *in vivo* adult [32–35] studies [36]. Iterative reconstruction will be particularly useful in low-dose CT of the urinary tract where image noise is typically high.

The next step in optimizing image quality in studies acquired at significantly reduced radiation dose is the ongoing development of advanced generations of iterative reconstruction such as model-based iterative reconstruction (MBIR), which is being developed by GE Healthcare. MBIR is a fully iterative reconstruction algorithm, which incorporates a physical model of the CT system into the reconstruction process to characterize the data acquisition process, including noise, beam hardening, and scatter. However, due to limitations in computing power and reconstruction technology, model-based iterative approaches have not been practical for commercial CT scanners until recently as reconstruction times had been exceedingly long.

## 4. Detection of Residual Urinary Calculi and Their Fragments

Studies which initially compared noncontrast CT of the urinary tract with plain radiography, ultrasound, and intravenous urography found significantly increased detection of urinary calculi with noncontrast CT leading to its immediate adoption as the imaging investigation of choice in the setting of suspected urinary tract calculi [1, 37, 38]. A recognized limitation in studies, which report the diagnostic performance of noncontrast CT of the urinary tract, is the choice of gold standard investigation upon which sensitivity and specificity calculations are based. In many studies, the identification of false negative cases is dependent on additional calculi being subsequently identified with clinical followup, urography, and/or endoscopy in some cases. This may potentially lead to an inappropriately low detection of false negative cases and consequently a spurious increase in the reported sensitivity of noncontrast CT [39].

In relation to the task of completely excluding all residual stones and their small fragments after endourological treatment, CT has recognized limitations. CT has a limited spatial resolution with typical pixel dimensions in the *x*-*y* axis measuring 0.7–1 mm in diameter; a typical slice reconstruction thickness of 2–5 mm further reduces spatial resolution along the *z*-axis and thereby obscuring small calculi. Reducing the radiation dose imparted during noncontrast CT of the urinary tract also appears to reduce the spatial threshold at which calculi become invisible [39, 40]. Currently, the best available experimental evidence indicating a size threshold for accurate exclusion of calculi is provided by a cadaveric study by Jin et al. who imaged three to five renal stones measuring 2.0–4.0 mm that were placed in 14 cadaveric human kidneys [39]. The investigators found poor detection of calculi measuring less than 2 mm on both low-dose (29% sensitivity) and conventional-dose (47–59% sensitivity) CT images. Jin et al. conclude that many previous trials examining the diagnostic performance of noncontrast CT of the urinary tract may overestimate the sensitivity and negative predictive value for detection of small urinary calculi measuring less that 2 mm in size, but calculi measuring greater than 4 mm were identified in a much higher proportion of cases (95–100% sensitivity) for both low-dose and conventional-dose CT [39]. 

Other authors have found reductions in sensitivity and specificity for detecting small calculi (<3 mm) when low-dose CT techniques were employed [14–24, 40, 41]. Although results vary according to the extent of dose reduction achieved, the majority of studies suggest that confident exclusion of calculi measuring >4 mm in diameter is possible with most low-dose imaging protocols [14–24, 40, 41].

## 5. Characterization of Urinary Calculi

The attenuation value of different subtypes of renal calculi overlaps greatly on conventional single-energy CT datasets [42], but their attenuation values differ significantly when imaged with high- and low-energy CT. Uric acid stones, which are predominantly composed of low-molecular-weight elements (oxygen, carbon, and nitrogen), have different X-ray attenuation properties at high- and low-energy CT compared with other types of renal calculi such as calcium oxalate, hydroxyapatite, or cysteine stones. These stones are composed of high-molecular-weight elements (phosphorus, calcium, and sulfur) and therefore, as a consequence, will have a higher Hounsfield unit value at lower-energy CT.

**Figure 2:**
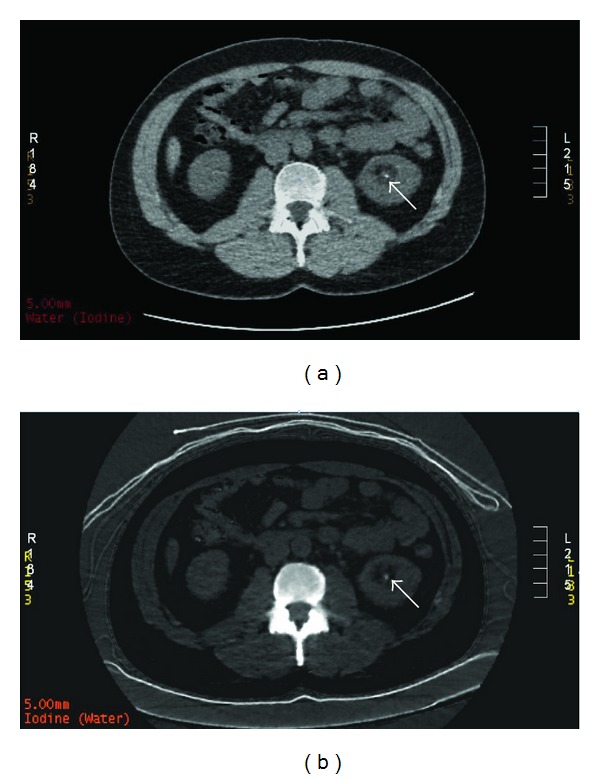
A 34-year-old male presenting with left flank pain. Axial dual energy noncontrast CT. (a) shows a 3 mm calculus in the left renal pelvis on iodine and (b) water-based attenuation. As the calculus is visible on both imaging techniques, this indicates high-molecular-weight elements. This calculus proved to be predominantly calcium based.

**Figure 3:**
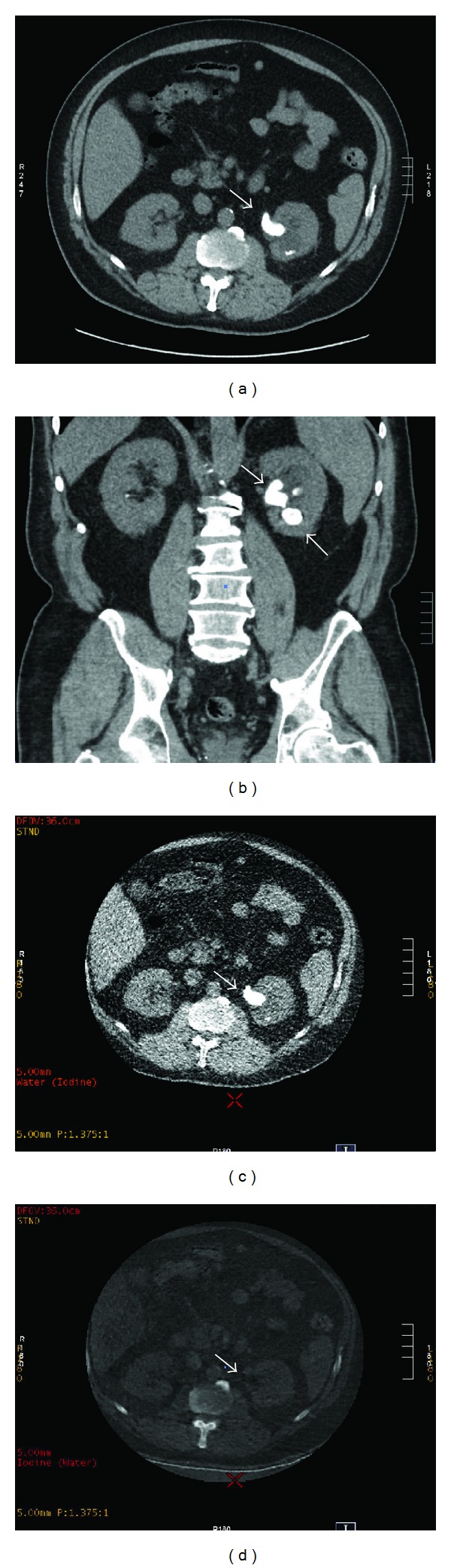
A 42-year-old male with a history of renal calculi presenting with left flank pain. Noncontrast CT for the assessment of renal calculi in axial (a) and coronal (b) reformats. These images clearly show a large calculus in the left renal pelvis extending into the upper ureter as well as a second large calculus in the lower pole of the left kidney (arrows). Axial dual energy noncontrast CT images in the same patient showing iodine- (c) and water- (d) based attenuation. The calculus is visible on the iodine-attenuated image (c) and is unchanged when compared to the standard CT. However, the water-attenuated image (d) shows “dropout,” and the calculus is no longer visible. This indicates the presence of low-molecular-weight elements. Post removal, this stone proved to be predominantly composed of urate.

This characteristic difference in attenuation with dual-energy imaging (i.e., contemporaneous CT scanning at 80 and 120−140 Kv) may potentially allow accurate determination of stone composition which may facilitate more appropriate management in patients with uric acid (UA) containing calculi who may benefit from medical management and those with cystine and certain calcium stones which may be more resistant to shock wave lithotripsy [43] (see Figures 2–3).

Studies involving dual-energy CT have proven the accuracy of this technique in both *ex* and *in vivo* studies [44–46]. 

Accuracy of dual-energy imaging appears however to also be dependent on stone size. In an *in vivo* study conducted by Manglaviti et al., where there was disagreement between the chemical composition analysis in urinary tract calculi determined by dual-energy CT and the actual composition determined by crystallography, the stone diameter in each case was less than 1 cm; in each discrepant case, mixed uric acid and hydroxyapatite calculi were misclassified as cystine and hydroxyapatite on dual-energy CT [46].

It should also be acknowledged that at present, dual-energy imaging is associated with higher doses of ionizing radiation when compared with single-energy CT [47]. Work is currently underway to reduce radiation exposure associated with dual-energy imaging, however, and Thomas et al. report successful differentiation between calcific, uric acid, and cystine containing calculi with an effective dose comparable to intravenous pyelography in nonobese patients (2.7 mSv) [45]. Targeted dual-energy scanning of calculi can be incorporated into a standard noncontrast CT scan in a dose-efficient way as follows: a single-high-energy low-dose scan of the entire urinary tract can be performed followed by targeted low-energy scanning of the areas where calculi are located. Use of imaging strategies such as these has been shown to decrease effective dose, but early results indicate a sustained increase in dose (59%) when compared with low-dose single-energy CT alone [48].

Another approach is using CT to determine the relative fragility of calcium oxalate stones. Zarse et al. describe the use of micro-CT to delineate the internal structure of such calculi, as opposed to simple Hounsfield measurements, in the assessment of calculus suitability for shockwave lithotripsy. Their research showed that patients with calcium oxalate stones of homogenous composition were less likely to benefit from lithotripsy treatment than those with calculi displaying visible internal structure on micro-CT. The latter group of patients was found to have stones that were amenable to medical treatment as opposed to the lithotripsy-resistant calculi with homogenous internal architecture. The authors go on to suggest that pretreatment CT can be used to assess calculus fragility and that it is stone morphology, rather than X-ray attenuation, that better correlates with overall fragility [49].

## 6. Conclusion

As a result of CT dose reduction measures that have been outlined in this paper, a cancer risk that was small to begin with is being systematically reduced [50]. Average radiation exposures associated with CT scanning of the urinary tract for urinary tract calculi are likely to reduce further and may eventually reach doses similar to those currently encountered in plain radiography with the help of iterative image reconstruction and other techniques. The statistical risks associated with performing a clinically indicated CT will therefore be reduced, but individual justification for performing CT will still be required.

Noncontrast CT remains the best imaging modality for the detection of urinary calculi and it has a high negative predictive value in excluding calculi, measuring greater than 4 mm. Unfortunately, its spatial resolution, particularly when low-dose CT protocols are instituted, indicates that it is not suitable for completely excluding submillimeter calculi and small stone fragments in patients who are postendourological stone removal.
